# An Investigation of the Glucose Monitoring Practices of Nurses in Stroke Care: A Descriptive Cohort Study

**DOI:** 10.1155/2013/715802

**Published:** 2013-08-25

**Authors:** Elizabeth Ann Laird, Vivien E. Coates, Assumpta A. Ryan, Mark O. McCarron, Diane Lyttle, David Chaney

**Affiliations:** ^1^School of Nursing, University of Ulster, Northland Road, Londonderry BT487JL, UK; ^2^School of Nursing, University of Ulster and Western Health and Social Care Trust, Londonderry BT476SB, UK; ^3^Consultant Neurologist, Department of Neurology, Altnagelvin Hospital, Western Health and Social Care Trust, Londonderry BT476SB, UK

## Abstract

Glucose derangement is commonly observed among adults admitted to hospital with acute stroke. This paper presents the findings from a descriptive cohort study that investigated the glucose monitoring practices of nurses caring for adults admitted to hospital with stroke or transient ischaemic attack. We found that a history of diabetes mellitus was strongly associated with initiation of glucose monitoring and higher frequency of that monitoring. Glucose monitoring was continued for a significantly longer duration of days for adults with a history of diabetes mellitus, when compared to the remainder of the cohort. As glucose monitoring was not routine practice for adults with no history of diabetes mellitus, the detection and treatment of hyperglycaemia and hypoglycaemia events could be delayed. There was a significant positive association between the admission hospital that is most likely to offer stroke unit care and the opportunity for glucose monitoring. We concluded that adults with acute stroke, irrespective of their diabetes mellitus status prior to admission to hospital, are vulnerable to both hyperglycaemic and hypoglycaemic events. This study suggests that the full potential of nurses in the monitoring of glucose among hospitalised adults with stroke has yet to be realised.

## 1. Introduction

Nurses have a substantial role in the assessment and monitoring of adults admitted to hospital with stroke. Research has indicated that nurse-led interventions, including enhanced monitoring and management of glycaemia in the acute phase of stroke, mediate towards a positive clinical outcome after stroke [1]. Diabetes mellitus is a major risk factor for stroke, and impaired glucose tolerance and impaired glucose metabolism, intermediate conditions to type 2 diabetes, are contributing risk factors [2]. Whilst up to one-third of adults admitted to hospital with acute stroke may have a history of diabetes mellitus, hyperglycaemia is also commonly observed among those with no such history [3]. Transient patterns of hyperglycaemia that revert spontaneously to normoglycaemic ranges within 48 hours are indicative of a stress or inflammatory response [4, 5]. In contrast, patterns of delayed hyperglycaemia and persisting hyperglycaemia are most likely explained by the presence of diabetes or prediabetes syndromes [6–11]. Glucose regulation after stroke is a complex area [12–15]. The focus of this paper is glucose monitoring practice in stroke care.

Internationally respected clinical guidelines [16–19] have consistently recommended that glucose is monitored when adults are admitted to hospital with stroke. A descriptive cohort study was conducted that examined the extent to which glucose derangement was managed and treated in routine stroke care [20]. The main findings indicated that glycaemic status was undermonitored, hyperglycaemia was undertreated, and screening practices for undiagnosed diabetes mellitus could be improved. Hyperglycaemia (glucose ≥ 7.8 mmol/L) affected 24–34% patients over the first five days. Analysis of variance testing of highest glucose measurements from day 1 to day 5 demonstrated no significant effect (*P* = 0.339), thus indicating the persisting nature of hyperglycaemia. In additional analysis we found a 5% risk among the cohort for hypoglycaemic (glucose < 3.5 mmol/L) events [21]. These hypoglycaemic events were not associated with a history of diabetes mellitus.

Of all professions, nurses have the pivotal role in interpreting, reporting, and managing glucose derangement. They are the prime decision makers in hospital settings, in relation to when to initiate point of care glucose monitoring and the frequency and duration of such monitoring. Glucose derangement has an adverse impact on conscious level and cognitive functioning. It is also associated with impaired immunity, higher infection rate and fluid balance deregulation [22], and poorer clinical outcome after stroke [3, 4, 6–9]. If glucose monitoring practice in stroke care is found to be suboptimal, this warrants further research. All too often, clinical decision making is embedded in ritualistic ways of working and the socioculture of the hospital setting [23]. Consequently, the extent to which diabetes mellitus status, glucose results, and the care setting are influencing monitoring practice needs to be exposed.

The overall aim of the study was to investigate the glucose monitoring practices of nurses caring for adults admitted to hospital with stroke or transient ischaemic attack. The objectives were to explore the association between diabetes mellitus status and the initiation and duration of glucose monitoring, the association between glucose results and glucose monitoring rates, and the association between care setting and glucose monitoring practice.

## 2. Materials and Methods

The design was a retrospective cohort study informed by the STROBE Statement Group guidance [24]. Retrospective designed studies provide a good indicator of actual practice as clinician behaviour is not moderated. Our findings should be assessed within the context of the sampling strategy, that is, a convenient sample of 112 consecutively admitted adults with confirmed ischaemic stroke events or primary haemorrhagic stroke, to one Health and Social Care Trust. The stroke clinical guidelines [17, 25] and research findings [4, 5, 8–11] available at the study time period were indicating that glucose should be monitored. 

Data were derived from an in-depth review of the medical and nursing records of adults (*n* = 112) with a confirmed diagnosis of acute stroke or transient ischaemic attack (*n* = 112) admitted to three district general hospitals in the time period of January 1st to April 15th, 2008. The data were extracted in the audit departments of each of the three hospitals. Data extraction was undertaken between November 1st and December 22nd, 2009. In January 2010, an independent auditor reviewed 5% of randomly selected records, confirming strong interrater reliability of 0.94–1.0 for continuous variables. 

The study was conducted in a large health and social care trust located in the United Kingdom. The trust serves a population of 300,000 people. The three district general hospitals in the Trust accepted stroke admissions at the time of data collection, and all had a recognised stroke unit. As the purpose of this study was to evaluate practice retrospectively, the decision was taken that a convenient sample of consecutively admitted adults with acute stroke or transient ischaemic attack was appropriate. 

A proforma tool, informed by the research and internationally respected stroke guidelines was developed in collaboration with stroke clinicians employed in the NHS Trust. The proforma tool was then tested out in a small pilot study of 15 adults, which informed some amendments to the proforma, mainly in relation to the sequencing of the data collection items. The medical and nursing records of adults admitted to hospital with acute stroke of transient ischaemic attack provided the data sources. Data were collected to determine the baseline characteristics of the sample, and these included age, gender, risk factors for stroke, comorbidities, and stroke subtype. 

The chief variables were glucose levels in mmol/L at baseline and in each of the first 5 days of hospital, the number of times each day that point of care glucose monitoring was undertaken and the number of days that point of care glucose monitoring was undertaken. In this study, point of care glucose monitoring refers to registered nurses undertaking capillary finger prick testing of adults, with the use of lancing devices, testing strips, and small portable calibrated glucometers. Glucose levels were determined by examination of both laboratory test results that indicated the glucose level and point of care glucose monitoring records. The frequency and duration of point of care glucose monitoring practice were determined from point of care glucose monitoring records. The chief outcome measure was rates of point of care glucose monitoring in each of the first five days. A secondary outcome measure was the rate of  HbA1c testing undertaken at any time during the in-hospital episode. Care setting was determined by the type of hospital ward the adult was treated in during the first 5 days, for example a stroke unit, an emergency assessment unit, or a general medical ward.

The cohort was divided into two groups, determined by prehospital diabetes mellitus status. This enabled comparisons of age, gender, risk factors for stroke, comorbidities, stroke subtype, and glucose level variables between the two groups, to detect for significant differences. This division also enabled comparisons in point of care glucose monitoring practice between the groups, to determine if there was an association between diabetes mellitus status and point of care glucose monitoring practice.

Following this, the cohort was divided into three groups, determined by which of the three hospital, the adult was admitted. This enabled comparisons of rates of admission to a stroke unit, point of care glucose monitoring practice, and HbA1c tests undertaken, to determine if care setting was influencing practice. 

Data were analysed using SPSS for Windows version 17 [26]. Descriptive statistics were employed to explore the characteristics of the cohort. Tests of normality were then undertaken on the distribution of continuous variables, for example, age and blood glucose levels. Nonsignificant results for the Kolmogorov-Smirnov statistic and visual inspection of the shape of distributions of continuous variables indicated normality and facilitated the use of parametric statistical tests. Chi-square tests were conducted to explore differences in gender, risk factors, and comorbidities between adults grouped by prehospital diabetes status. Independent sample *t*-tests were employed to assess differences in continuous variables, including age and glucose results between adults grouped by diabetes mellitus status. A chi-square test was undertaken in order to investigate the association between diabetes mellitus status and the initiation of point of care glucose monitoring. An independent samples *t*-tests were undertaken to compare the duration (number of days) and frequency (number of times glucose monitored each day) of point of care glucose monitoring between adults grouped by prehospital diabetes mellitus status. Pearson's product-moment correlation tests were undertaken to investigate the relationship between highest and lowest glucose results and the frequency at which adults received point of care glucose monitoring. Analysis of variance tests were utilised to explore differences in continuous variables between adults grouped by admission hospital.

Our target sample was a minimum of 100 adults aged 18 and above. A sample size of 100 or more was sufficient to generate power at 95% confidence intervals, and this allowed for the use of the standard 0.05 alpha level cutoff for indication of a significant result [27]. The research team was aware that a percentage of adults admitted to the hospitals with a provisional stroke diagnosis would later have that initial diagnosis overturned. In order to ensure a target minimum sample of 100 adults with confirmed stroke or transient ischaemic attack, the records of the first 150 adults, aged 18 and over, admitted with stroke to one of the three hospitals on and after January 1st, 2008, were reviewed. The first stage of that review was to ascertain if a new diagnosis of acute stroke or transient ischaemic attack was confirmed. The initial diagnosis of stroke was overturned in 38 of the 150 adults, and thus the final sample size was 112 adults. 

In accordance with research governance in the study time period, ethical approval was sought from the clinical research and audit department of the NHS Trust involved. The request was approved and the records pertaining to all adults admitted with stroke, to the three hospitals in the trust, were requested. All the records requested were made available. 

## 3. Results

Of the 112 adults admitted to hospital with stroke, 51 (46%) were men and 61 (54%) were women.  Table 1 provides baseline characteristics of the sample. The age ranged from 24 years to 99 years, with the mean age being 74 years. An independent samples *t*-test was conducted to compare age for those with and without a history of diabetes mellitus and found that adults with a history of diabetes mellitus were significantly older in age (mean 78 versus 73 years, *P* = 0.02). In total, 18 (16%) of the cohort had a history of diabetes mellitus, of which 16 had type 2 diabetes. Fifty-one (45%) of the cohort had a history of hyperlipidaemia; and 48 (42%) had hypertension. A chi-square test for independence (with Yates continuity correction) indicated a significant association between hypertension and history of diabetes mellitus status (*P* = 0.003). The vast majority of the cohort had experienced an ischaemic event; 95 (85%) had experienced an ischaemic stroke or transient ischaemic attack, and 17 (15%) had a primary haemorrhagic stroke. 

### 3.1. The Association between Diabetes Mellitus Status and the Initiation and Duration of Glucose Monitoring

Table 2 provides a comparison of glucose results and point of care glucose monitoring practices between adults with and without a diagnosis of diabetes mellitus before admission to hospital. Among the cohort, there was evidence that 95 (84%) had a test undertaken at time of admission to establish baseline glucose. Mean baseline glucose was 7.3 mmol/L with the range 2.0–16.1 mmol/L. An independent samples *t*-test was conducted to explore differences in baseline glucose between adults with and without a history of diabetes, and baseline glucose was significantly higher for those with a history of diabetes (mean 9.6 versus 6.8 mmol/L, *P* = 0.005). Differences in mean highest glucose between the two groups remained significant on days 2, 3 and 4. On day 5, differences were no longer significant (*P* = 0.14). The tests undertaken to establish baseline glucose were routine electrolyte profile (43%), laboratory plasma glucose test (5%) and point of care glucose test (52%). It was clear that a diagnosis for diabetes mellitus was strongly associated with opportunity to receive point of care glucose monitoring on day one (*P* < 0.01) and higher frequency of that monitoring (*P* < 0.01). In contrast, only 15% of adults without a history of diabetes mellitus received such monitoring on day 1. An independent samples *t*-test was conducted to compare number of days of regular point of care glucose monitoring for adults with and without a history of diagnosed diabetes. The difference was significant (mean 15.88 versus 1.74 days, *P* = 0.034). 

### 3.2. The Association between Glucose Results and Glucose Monitoring Rates

Thirty-two (28%) adults received regular point of care glucose monitoring for at least 24 hours. Point of care glucose monitoring was undertaken for an average of four days. The relationship between the highest glucose result and the frequency of point of care glucose monitoring on day 1 was investigated using Pearson's product-moment correlation coefficient. There was a moderate, positive correlation between the two variables (*r* = 0.447, *n* = 110, *P* < 0.0005), with higher glucose results associated with higher frequency of glucose tests. The relationship between lowest glucose result and the frequency of near glucose monitoring on day 1 was investigated using Pearson's product-moment correlation coefficient. No significant correlation between the two variables was found (*r* = −0.021, *n* = 94, *P* < 0.845). 

### 3.3. The Association of Care Setting with Glucose Monitoring Practice

Our data were derived from the medical records of adults consecutively admitted to three district hospitals within the one Health and Social Care Trust.  Table 3 provides a comparison of practice across the three hospitals. A chi-square test for independence indicated a significant association between admission hospital and opportunity for treatment in a stroke unit (*P* < 0.001). So whilst 97% of admitted adults to hospital B were treated in a stroke unit, only 27% of admitted adults to hospital A received stroke unit care. A one-way between groups analysis of variance was conducted to explore baseline glucose result across the three hospitals, and the difference across the three groups was not significant (*P* = 0.97). However, a chi-square test for independence indicated a significant association between admission hospital and the opportunity for point of care glucose monitoring on day one (*P* = 0.002) with adults admitted to hospital B most likely to be monitored. Rates of point of care glucose monitoring decreased on subsequent days across all three hospitals, and differences were no longer significant (data not presented). The majority of adults (70%) received the standard fasting glucose test, with patients admitted to hospitals B and C most likely to be tested, although the difference was not significant (*P* = 0.06). Nine (8%) of the cohort had evidence of HbA1c testing. 

## 4. Discussion

This paper presents an analysis of glucose monitoring practice among a cohort of adults admitted to hospital with acute stroke or transient ischaemic attack. Among our cohort, 18 (16%) of the adults had a history of diabetes mellitus. This is lower than the prevalence rate of 20% reported in an Austrian study that similarly included adults with both ischaemic and primary haemorrhagic strokes [28]. Consistent with other studies [7–11], glucose derangement and hyperglycaemia in particular were common in acute stroke patients. Glucose was largely undermonitored, particularly among those adults who had no history of diabetes mellitus. This finding compares with those from similar research from Europe [5] and the USA [29, 30]. Our study is the first that attempted to investigate in any depth the association between diabetes mellitus status and the initiation and duration of point of care glucose monitoring, the association between glucose results and point of care glucose monitoring rates, and the association between care setting and glucose monitoring practice. 

### 4.1. The Association between Diabetes Mellitus Status and the Initiation and Duration of Glucose Monitoring

Prehospital admission diabetes mellitus status was strongly associated with initiation of point of care glucose monitoring and higher frequency of that monitoring on day one. In addition, adults with a history of diagnosed diabetes received point of care glucose monitoring for a significantly longer duration of days than the remainder of the cohort. Whilst 84% of our cohort had baseline glucose testing on admission to hospital, it is of note that 16% did not. Given that diabetes mellitus is a major risk factor for stroke [2] and that glucose derangement impacts on neurological functioning, it would be anticipated that all adults on admission to hospital with suspected stroke or transient ischaemic attack would receive glucose testing promptly as standard routine care. It is noteworthy that the updated American Heart Association/American Stroke Association guideline [19] emphasises this point, by explaining that of the number of haematological, coagulation, and biochemistry tests that are recommended in the initial emergency evaluation of the patient, only the assessment of blood glucose must precede the initiation of intravenous lysis treatment. 

Thirty-two (28%) adults received regular point of care glucose monitoring by registered nurses for at least a duration of 24 hours. A history of diabetes mellitus prompted nurses to initiate the point of care glucose monitoring. In contrast, only 15 (16%) of the 94 adults without a history of diabetes mellitus received such monitoring. When clinical guidelines are stipulating that glucose should be monitored after stroke, this low percentage is concerning. Our findings suggest that, without sufficient monitoring, hyperglycaemia will remain an underdiagnosed problem. Glucose derangement has serious medical implications. It is therefore incumbent on nurses in stroke settings to comply with the main international stroke guidelines [18, 19] that are recommending that glucose is monitored in all the acute stroke patients. 

Overall, the average duration of point of care glucose monitoring was four days. Our findings indicate that adults with a history of diabetes were monitored for a significantly longer period of days. Research indicates that delayed and persisting patterns of hyperglycaemic are relatively common among stroke cohorts [3, 6, 8, 11]. Middleton et al.'s [1] research on the implementation of evidence-based treatment protocols to manage hyperglycaemia, fever, and swallowing dysfunction in the acute stroke phase is welcome. Their clustered randomised controlled trial was conducted across 19 acute stroke units in Australia, and findings demonstrated that the nurse-led interventions delivered improved clinical outcomes after discharge. The duration of the interventions was 72 hours. Our research has previously reported a persisting trend of hyperglycaemia up to and including the first five days [20] and, even more concerning, a 5% incident rate of hypoglycaemia with glucose < 3.5 mmol/L [21], not associated with history of diabetes mellitus or administration of hypoglycaemic agents. Internationally respected clinical guidelines [19] recommend that hypoglycaemia 3.3 mmol/L should be treated and hyperglycaemia maintained in the 7.8–10 mmol/L range. However, adults will miss opportunity for treatment, if glucose is not monitored. Continuous glucose monitoring is becoming more available and has been used successfully among a stroke cohort [31]. It is likely that in the future, continuous glucose monitoring may become the monitoring strategy of choice when adults are admitted to hospital with a new stroke diagnosis.

When rates of HbA1c testing were compared between the cohort groups by prehospital diabetes mellitus status, a lower percentage of adults without a history of diabetes mellitus was tested. HbA1c provides a useful indicator of glycaemic control over previous weeks and months. The UK Department of Health Advisory on Diabetes [32] has recommended that, in general, HbA1c of greater than or equal to 48 mmol/mol (6.5%), rather than fasting glucose or a glucose tolerance test, should be used to diagnose diabetes, unless the person is acutely symptomatic or there is a suspicion of type 1 diabetes. An adult with HbA1c result between 42 and 47 mmol/mol (6.0–6.4%) should be considered to be at high risk of diabetes. The authors recommend that all adults admitted with stroke, irrespective of prehospital diabetes mellitus status, should have HbA1c testing.

### 4.2. The Association between Glucose Results and Glucose Monitoring Rates

Higher glucose results were positively associated with higher frequency of glucose monitoring on day one of hospital admission. This result is not surprising, given that nurses in our study were mainly monitoring the glucose of those adults who had a history of diabetes mellitus. In contrast, when the relationship between lowest glucose result and the frequency of near glucose monitoring on day 1 was investigated, no significant association was found. Our previous research reported some delays in treatment of both hyperglycaemia [20] and hypoglycaemia [21], and these were associated with low glucose monitoring rates. Compliance to the recommendation that all acute stroke patients should have their glucose monitored should assist nurses to identify and manage glucose deregulation in a more timely manner. 

### 4.3. The Association between Care Setting and Glucose Monitoring Practice

Whilst baseline glucose was similar among adults grouped by admission hospital, we found a significant association between admission hospital and the opportunity for point of care glucose monitoring on day one. It was clear that patients admitted to hospital B, where 97% of patients were treated in a stroke unit, were more likely to be monitored. This finding supports the important role of stroke unit nurses in glucose monitoring practices, since they are more likely to work under stroke care pathways based on international guidelines [1, 33].

In summary, glucose derangement remains an underdiagnosed problem in acute stroke care, affecting adults with and without a history of diabetes mellitus. Despite internationally respected stroke guidelines recommending that glucose is monitored in all adults with acute stroke, our research would indicate that it is still not a widespread practice in many clinical settings. Adults with acute stroke admitted to hospitals which can ensure stroke unit care are more likely to have their glucose monitored. 

## 5. Conclusion

Glycaemic control is a subject of significant interest in acute stroke. We conducted a retrospective cohort study to investigate the association between diabetes mellitus status and glucose monitoring practices, the association between glucose results and glucose monitoring rates, and the association between care setting and glucose monitoring practice. This study suggests that the full potential of nurses in the monitoring of glucose among hospitalised adults with stroke has yet to be realised. Our findings suggest that a history of diabetes mellitus is not an appropriate criterion to guide glucose monitoring practices in acute stroke care. Our finding that glucose was undermonitored particularly among adults with no history of diabetes mellitus demonstrates that much work remains to be done to adapt clinical practice to the recommendations of the international stroke guidelines. Stroke unit nurses have an important role in the effort to enhance glucose monitoring practice. 

## Figures and Tables

**Table 1:** Characteristics of the cohort and comparison between adults grouped by history of diabetes mellitus and no history of diabetes mellitus.

Characteristic	Total *n* (%)	History DM *n* (%)	No history DM *n* (%)	*P* value
Demographics	112 (100)	18 (16)	94 (84)	
Age*, mean ± SD (range)	74 ± 13 (24–99)	78 ± 6.5	73 ± 14.2	0.02
Women	61 (54%)	8 (44)	53 (56)	0.5
Risk factors/comorbidities				
Atrial fibrillation	22 (20%)	3 (17)	19 (20)	
Previous stroke or TIA	41 (37%)	6 (33)	35 (37)	0.96
Hyperlipidaemia	51 (45%)	12 (67)	39 (41)	0.08
Hypertension^†^	48 (42%)	14 (78)	34 (36)	0.003
MI or angina	27 (24%)	7 (39)	20 (21)	0.19
Valvular heart disease	4 (4%)	0 (0%)	4 (4)	
Current smoker	13 (12%)	0 (0)	13 (14)	
Stroke type after scan				
Ischemic stroke/TIA	95 (85%)	16 (89)	79 (84)	
Primary haemorrhagic stroke	17 (15%)	2 (11)	15 (16)	

DM: diabetes mellitus, TIA: transient ischaemic attack, and MI: myocardial infarction. An independent sample *t*-test compared *age across the two groups, and there was a significant difference. A chi-square test for independence (with Yates continuity correction) found a significant difference in rates of ^†^hypertension between the two groups. *P* values < 0.05 indicate significance. If counts in cells were less than five, statistical analysis was not conducted.

**Table 2:** Glucose results (mmol/L) and glucose monitoring practice and comparison between adults grouped by history of diabetes and no history of diabetes mellitus.

Variable	Total *n* (%)	History DM *n* (%)	No history DM *n* (%)	*P* value
Adults in hospital day 1, *n* (%)	**112 (100)**	**18 (16)**	**94 (84%)**	
Baseline glucose mmol/L, m ± SD (*r*)	7.3 ± 2.6 (2.0–16.1)	9.6 ± 3.6	6.8 ± 2.1	0.005
Highest glucose mmol/L, day 1, m ± SD (*r*)	7.8 ± 3 (2.4–17)	10.3 ± 3 (4.8–16.1)	7.2 ± 2 (2.4–17.1)	0.002
Lowest glucose mmol/L, day 1, m ± SD (*r*)	6.8 ± 2 (2.4–14.7)	7.9 ± 3 (4.3–13.5)	6.5 ± 2 (2.4–14.7)	0.067
PGM conducted day 1	32 (28)	18 (100)	14 (15)	0.00
Adults in hospital day 2, *n* (%)	**105 (100)**	**17 (16)**	**88 (84)**	
Highest glucose mmol/L, day 2, m ± SD (*r*)	7 ± 3 (4–24)	9.4 ± 3 (4.3–14.7)	6.3 ± 3 (4.3–24.1)	0.001
PGM conducted day 2	21 (20)	16 (94)	5 (6)	
Adults in hospital day 3, *n* (%)	**98 (100)**	**14 (14)**	**84 (86)**	
Highest glucose mmol/L, day 3, m ± SD (*r*)	7.7 ± 3.5 (2.9–19)	9.1 ± 2.4 (5.7–12.9)	7.1 ± 4 (2.9–19)	0.06
PGM conducted day 3	20 (20)	14 (100)	6 (7)	
Adults in hospital day 4, *n* (%)	**89 (100)**	**12 (13)**	**77 (86)**	
Highest glucose mmol/L, day 4, m ± SD (*r*)	7.6 ± 3.6 (3.8–19)	9.7 ± 3 (5.9–18)	6.8 ± 3 (3.8–19)	0.019
PGM conducted day 4	18 (21)	12 (100)	6 (8)	
Adults in hospital day 5, *n* (%)	**81 (100)**	**11 (13)**	**70 (86)**	
Highest glucose mmol/L, day 5, m ± SD (*r*)	8.1 ± 3.6 (3.4–18.5)	9.5 ± 2.6 (6.8–13.9)	7.5 ± 4 (3.4–18.5)	0.14
PGM conducted day 5	12 (15)	8 (73)	4 (6)	
Number of days glucose monitored, m ± SD (*r*)	3.9 ± 14.7 (0–100)	15.9 ± 24.78	1.74 ± 10.9	0.034
HbA1c test undertaken	9 (8)	4 (22)	5 (5)	

DM: diabetes mellitus, m: mean, SD (*r*): standard deviation (range), and PGM: point of care glucose monitoring. *t*-tests indicated that adults with history DM had significantly higher glucose at baseline and highest glucose recordings on day 1, day 2, and day 4. Chi-square test for independence (with Yates continuity correction if 2 by 2 table) found that history of DM was significantly associated with higher frequency of testing on day 1 and longer duration of testing in terms of numbers of days that glucose was monitored. *P* values < 0.05 indicate significance. If counts in cells were less than five, statistical analysis was not conducted.

**Table 3:** Glucose monitoring practice and between adults admitted to hospitals A, B, and C.

Variables	Total *n* (%)	Hospital A *n* (%)	Hospital B *n* (%)	Hospital C *n* (%)	*P* value
Adults admitted, *n* (%)	112 (100)	59	31	22	
History DM	18 (16)	8 (14)	4 (13)	6 (27)	
Treatment in stroke unit**	68 (61)	27 (46)	30 (97)	11 (50)	0.00
Baseline glucose mmol/L, m ± SD	7.3 ± 2.6	7.3 ± 2.05	7.3 ± 2.75	7.4 ± 3.16	0.97
PGM^††^ conducted day 1	32 (28)	10 (17)	15 (50)	7 (32)	0.005
Fasting glucose tested	78 (70)	34 (63)	26 (83)	18 (82)	0.06
HbA1c undertaken	9 (8)	6 (10%)	2 (7%)	1 (5%)	

DM: diabetes mellitus, m: mean, SD: standard deviation, and PGM: point of care glucose monitoring. Between groups analysis of variance [ANOVA] tests were conducted to compare baseline glucose (mmol/L) across the 3 hospital groups. Chi-square tests were conducted to compare treatment variables across the 3 groups. Adults admitted to hospital B were significantly more likely to receive treatment in a **stroke unit and have ^††^PGM on day 1. *P* values < 0.05 are significant. If counts in cells were less than five, statistical analysis was not conducted.
